# Short-Term Energy Demand Forecast in Hotels Using Hybrid Intelligent Modeling †

**DOI:** 10.3390/s19112485

**Published:** 2019-05-31

**Authors:** José-Luis Casteleiro-Roca, José Francisco Gómez-González, José Luis Calvo-Rolle, Esteban Jove, Héctor Quintián, Benjamin Gonzalez Diaz, Juan Albino Mendez Perez

**Affiliations:** 1 Department of Industrial Engineering, University of A Coruña, A Coruña 15280, Spain; jose.luis.casteleiro@udc.es (J.-L.C.-R.); jose.rolle@udc.es (J.L.C.-R.); esteban.jove@udc.es (E.J.); hector.quintian@udc.es (H.Q.); 2 Department of Computer Science and System Engineering, Universidad de La Laguna, La Laguna 38200, Spain; 3 Department of Industrial Engineering, Universidad de La Laguna, La Laguna 38200, Spain; jfcgomez@ull.edu.es (J.F.G.-G.); bgdiaz@ull.edu.es (B.G.D.)

**Keywords:** energy forecast, artificial neural network, hybrid modeling, support vector regression, hotel, tourism

## Abstract

The hotel industry is an important energy consumer that needs efficient energy management methods to guarantee its performance and sustainability. The new role of hotels as prosumers increases the difficulty in the design of these methods. Also, the scenery is more complex as renewable energy systems are present in the hotel energy mix. The performance of energy management systems greatly depends on the use of reliable predictions for energy load. This paper presents a new methodology to predict energy load in a hotel based on intelligent techniques. The model proposed is based on a hybrid intelligent topology implemented with a combination of clustering techniques and intelligent regression methods (Artificial Neural Network and Support Vector Regression). The model includes its own energy demand information, occupancy rate, and temperature as inputs. The validation was done using real hotel data and compared with time-series models. Forecasts obtained were satisfactory, showing a promising potential for its use in energy management systems in hotel resorts.

## 1. Introduction

The tourism sector has become a very important sector, and its activity plays a key role in the development of regions and the use of raw materials and energy that influence the environment. According to the World Tourism Organization (UNWTO), tourism accounts for almost 10% of the World Gross Domestic Product (GDP) and is responsible for 1 out of every 10 jobs in the world. The tourism sector, worldwide, ranks fourth in terms of export volume, after fuels, chemicals, and food. The world tourism grew, according to the UNWTO, by 7% in 2018 compared to the same period in the previous year, which is the highest growth in international tourist arrivals since 2010.

After transportation, the hotel industry is the activity with the most energy consumption. The average hotel consumption ranges can be between 450 and 700 kWh/m${}^{2}$ per year, corresponding to over 60% of electricity. However, these values are quite variable depending, among other things, on the climatic conditions and the category of the hotel [1]. Any scenario for the study of energy consumption and the incorporation of renewable energies in the tourism sector should be mainly focused on the study of the hotel sector. In particular, in tourism-based economies, there are several conditions, such as a high number of visitors, a long average stay, and a high percentage of quality hotels, that make the hotel sector an important element for energy demand profiles. In practice, the entire area where the hotel is located is affected by its activity. On the other hand, most overnight stays are made in hotels with 4 or more stars. Therefore, the high standing of the hotel sector with its particular profile plays a central role in tourism development and energy demand profile.

To achieve the objectives of reducing consumption, efficient energy measures must be implemented, and renewable energy production technologies must be introduced. In a study carried out in a large hotel in the subtropical zone of the coast of Queensland in Australia, it is concluded (with 2004 prices) that renewable energy sources by themselves are neither technically nor economically viable, but they will be if a connection to the electricity grid exists [2].

Many studies have focused on the analysis of energy performance in hotels. In a study by Dang & Burnett [3], they showed the difficulties in assessing energy performance in hotels. The study showed that performance indexes defined for other types of commercial buildings are not adequate for assessing energy performance of hotel buildings. In other works, models to correlate the energy demand with different hotel parameters were proposed. Most common parameters were the occupancy, the number of beds, and the number of workers. However, results suggest that it is not easy to find a model that suits all hotels. For example, a study by Papamarcou and Kalogirou [4] showed that for a hotel in Cyprus, there was a good quadratic correlation between electricity consumption and the number of guests, but another author, Priyadarshini et al. [5], did not find that same relationship in hotels in Singapore. Another example is the work of Cabello and collaborators [6], who apply an energy efficiency index that considers the outdoor ambient temperature, and that was implemented in two hotels in Cuba, achieving a 10% reduction in electricity consumption. Therefore, the definition of efficient numerical indexes for energy performance is a task that has not yet been resolved, and which depends on the type of hotel, location, climate, etc.

The need for reliable tools for energy forecast seems clear. This will allow for a better planning of hotel activities and management of energy resources. Consequently, industrial activity could be developed more sustainably and respectfully in an environmentally aware way, thus reducing the ecological footprint. In addition, energy demand management helps to achieve self-sufficiency and cost effectiveness. In this sense, for instance, the Hilton Worldwide has implemented its LightStay platform developed to calculate and analyze the environmental and societal impact of every Hilton Worldwide branded hotel [7]. Presently, the LightStay can track historical energy and weather data to forecast future energy consumption levels and predict the impact of performance on cost, to take corrective actions to influence future performance. These kinds of systems are commonly integrated in the Building Management System (BMS) that will propose the best management of 6 resources in the hotel to guarantee the efficient use of energy resources. The success of these systems depends on the availability of accurate forecasting models.

From a methodological point of view, different approaches have been used to predict energy consumption in hotels. Forecasting can be short-term (1 h to 1 week), medium-term (1 week to 1 year), or long-term (above 1 year). This paper focuses on short-term forecasting. For this, several methods have been proposed ranging from similar historical days approach, expert systems, regression models, time-series models, and artificial intelligence methods. Similar-day approach is based on predicting load values based on the information from previous days with similar characteristics. Regression is a statistical technique whose aim is to model the relationship of a dependent variable (energy load) and one or more independent variables (weather conditions, occupancy, etc.). Time series is one of the most-used techniques and is based on using past values to predict future load values. The most-used time-series models are ARMA (Auto-Regressive Moving Average) and ARIMA (Auto-Regressive Integrated Moving Average) which use time and load as input parameters. ARIMAX (Auto-Regressive Integrated Moving Average with eXplanatory variable) is a variation of these models that includes exogenous variables [8,9,10]. An extension to ARIMA that supports the modeling of the seasonal component of the data is the Seasonal ARIMA (SARIMA), which has been proven to have better performance when this seasonal effect is present [10].

The rapid growth of available computational power and the development of new artificial intelligence methods has made possible the extensive use of these techniques for the prediction of both energy generation and consumption. Many examples of Artificial Intelligence (AI) based on predictive models can be found in the recent literature [11,12,13,14,15,16,17,18,19]. In [20], a model based on an Artificial Neural Network (ANN) to forecast building energy consumption is proposed. Recently, Muralitharan et al. [21] compared different neural networks for energy demand prediction in a smart grid. In the same sense, other authors [22,23], used fuzzy logic to calculate the load curve of a residential consumer. In [24], the authors propose a deep-learning framework to forecast electricity demand based on a Long Short-Term Memory network. They incorporated a moving window-based technique to improve the results compared to other neural network-based structures. In [25] the accuracy and generalization capabilities of some machine-learning-based techniques for predicting hourly Heating, Ventilation and Air Conditioning (HVAC) energy consumption of a hotel building is studied. In particular, a model based on Deep Highway Networks (DHN), Extremely randomized Trees (ET), and Support Vector Regression (SVR) were proposed for predicting hourly HVAC energy consumption of a hotel. No significant differences were found between them. Other proposals that also use machine learning can be found in [26,27,28,29,30,31].

This work is focused specifically in hotel modeling. The main contribution of our work is the proposal of an intelligent model to efficiently predict the energy demand in a hotel. The hotel energy consumption in a 24 h horizon is provided. We implemented the model in a luxury 5-star hotel on the island of Tenerife, Spain. The advantage of the model is that first, a sequential hybrid structure is considered. Thus, for each prediction time instant, an intelligent hybrid model is obtained. The hybrid nature increases the accuracy of the model as it can efficiently incorporate the nonlinear nature of the process. Secondly, the model can easily include information of the main variables affecting the load demand in the hotel. The resulting model is validated and compared with a time-series model based on the ARIMAX structure and three SVM-based methods.

The organization of this paper is structured as follows. First, an introduction to energy management systems is presented. In the next section, the methods used in the work are described. Then, the model for the prediction of daily energy demand is proposed. Finally, in the Results and Discussion Section, the main results are presented, in which the real energy demand and the predicted demand are compared and analyzed.

## 2. Energy Management in the Hotel Industry

The high energy demand associated with tourism activities makes necessary the implementation of efficient energy management strategies to reduce their environmental impact and to minimize the cost of operation. The activities that involve more energy consumption in the hotel industry are HVAC, lighting, domestic hot water (DHW), swimming pools heating, kitchens, and leisure activities. According to UNWTO, air conditioning consumes most of the demanded energy (with typical values higher than 40%). Energy consumption in DHW systems is around 15% of total energy, while lighting entails a much more variable energy demand (15% to 40%), depending on the type of hotel.

The complexity of this structure with a high variety of facilities and services makes it necessary to use specific tools for energy management in hotels. This type of tool, apart from fulfilling the objective of improving the profitability of the installation, will allow progress towards the environmental sustainability of the sector. The centralized control systems or BMS, allow management of the different elements of the installation (HVAC, DHW, etc.). A BMS is based on a computerized system that defines the preset requirements of the whole building and controls the different systems to take the building to the desired state of operation. A BMS needs information from different sensors to take decisions. Part of this information can be predictions of the future evolution related to building facilities, weather conditions, energy generation or consumption, etc.

Additional difficulties are related to the introduction of renewable sources as energy generators. This new scenario, where hotels become prosumers, requires advanced control systems that allow the integration of this energy resource into the system in the most efficient manner. For this purpose, EMS (Energy Management Systems) arise. These systems are used for the monitoring, control, and optimization of the generation, transmission, and distribution of energy and, also, the management of microgrids. EMS (that can be integrated as a module of the BMS) analyze the behavior of the network components and make decisions about their efficient management. The main functions in isolated systems are related to ensuring the supply of energy to the load in all conditions, ensuring maximum use of the renewable source, minimizing the cost of energy (COE), protecting the components to avoid damage by overload, and guaranteeing the stability of the power system. For systems connected to the network, these functions include the control of the flow of energy to and from the network (generally aimed at reducing the flow of energy to the grid and improving self-consumption), monitoring the network, displacement of peaks in the curve of cargo and the management of the use of periods of low tariffs [32].

There are many studies where different strategies for energy management systems are collected. Many of them are based on the application of optimization techniques with linear programming [33] and with predictive control [34]. All these studies propose strategies to control the flow of energy between various generation systems, storage systems, loads, and the network. Different proposals based on AI techniques have also emerged to address this problem [35]. Among them, techniques based on fuzzy logic have been very successful in their application [36]. In the particular case of hotels, there are also examples of systems for advanced energy analysis and management such as those proposed in [6]. In this paper, a discussion about the indicators used for the prediction and control of electricity consumption in hotels is done and a new indicator proposal is made to detect more efficiently overconsumption or bad practices in energy management. In [37] a proposal for decision-making based on fuzzy logic is presented for the evaluation of energy-saving technologies in luxury hotels. Other works have focused on the development of control algorithms for the different services or activities of the hotel. In [38], nonlinear predictive control improved energy savings in hotel rooms. Another application is presented in [39] where the development of a control algorithm for the efficient operation of the cold plant in a hotel in a tropical area is addressed. The results in both cases highlight the importance of advanced control algorithms to improve energy savings and efficiency in hotels.

In the design of energy management systems, new advanced methods are based on the use of generation and/or load forecasts. In the work by Riverón et al. [40], an advanced energy management system to control the power flow in a hybrid generation energy system connected to grid and with energy storage is presented. One of the features of this proposal is the use of predictions as inputs to the EMS. This produces a significant increase in the performance of the system. Thus, the availability of reliable predictions will be necessary in advanced management systems to improve the overall system efficiency. The present work tries to contribute to this by proposing an intelligent prediction model to forecast the hotel load.

## 3. Methods

### 3.1. Data

In the Canary Islands, the tourism sector represents around 35% of GDP and generates about 40% of employment [41]. On the island of Tenerife, foreign tourists staying on the island recorded an increase of +11% in 2016 and the average duration of the stay of the tourism hosted was 7.54 days. Until July 2016, 68% of the lodged did so in hotels, compared to 32% who were extra-hoteliers. According to the number of hotel places in Tenerife, 74% of places have 4 or more stars (information obtained from the Canary Islands Statistics Institute). As can be concluded from these data, the hotel sector has a key importance in tourism development and hence in the economic sustainability of the island.

The methodology used to model the energy load in hotels is based on the use of real data from hotel resorts. The same data will be used to obtain a hybrid model based on intelligent techniques and to develop time-series models. The work presented here is based on data kindly provided by a luxury 5-star hotel, in the south of Tenerife, Canary Islands (Spain), located on the Atlantic Ocean (28.100${}^{\circ }$ N, 15.400${}^{\circ }$ W). These data consisted of demand of electricity, daily mean temperature, and occupation rate during a year recorded from 1 November 2016 to 31 October 2017 with a sample rate of one sample/hour).

**Figure 1:**
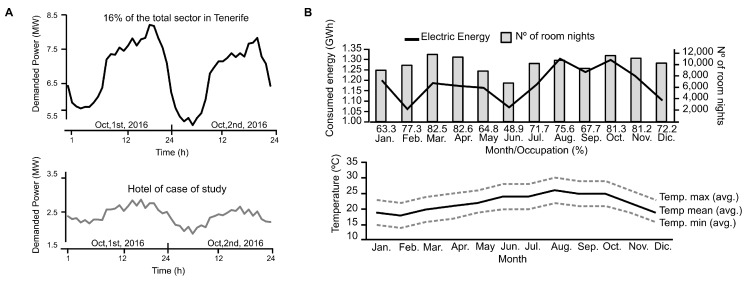
Energy and occupancy in 2017 in the hotel under study. (**A**) Power demand profile in hospitality sector. Elaborated with information provided by ENDESA and a luxury 5-star hotel. (**B**) Energy consumed and occupancy in 2017 in the hotel, and exterior temperature variation along 2017 from the nearest meteorological station, i.e., at the Tenerife South Airport (GCTS) (Source www.wunderground.com).

The daily power demand of the hospitality sector and a single hotel (our case of study) is shown in Figure 1A. The figure shows that demand profile of the hotel is quite similar to the hospitality sector. Consequently, the hotel selected for this work is a representative example of the demand of the tourism sector in Canary Islands.

The types of energy sources used in the hotel are: electricity from the grid, gas-oil, and thermal solar. However, the main consumed energy comes from the electric energy from the power grid. Regarding the consumption of electrical energy, Figure 1B shows the occupancy percentage of the hotel, the average outside temperature, and at the same time, the electrical energy consumed during 2017. From these data, we can estimate that on average a room night spends 118 kWh of electrical energy (or 144 kWh in total taking account the rest of energy sources).

### 3.2. ARIMAX Modeling

**Figure 2:**
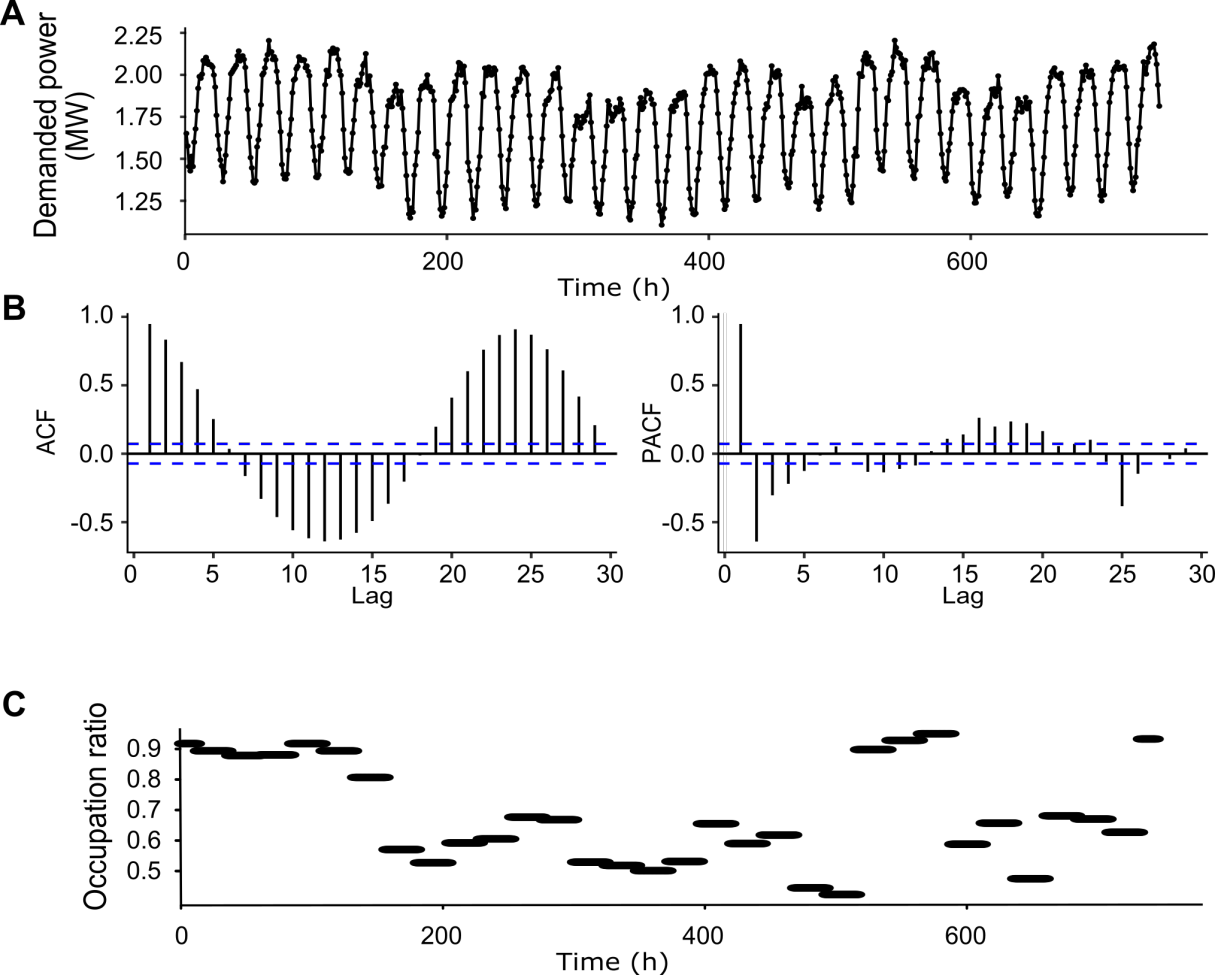
Demanded power by a hotel. (**A**) Demanded power by a hotel for 31 days. (**B**) Auto-Correlation Function and Partial Auto-Correlation Function of demanded power in (**A**); dashed blue lines indicate significance bounds. (**C**) Occupation ratio of the hotel during same period.

ARIMAX models are obtained for the time series of the demanded power (Figure 2A) by a hotel including the occupation (Figure 2C) as an exogenous variable. Ambient temperature values were not included as only daily mean and not hourly temperature is available. The first step is to study if the time series is stationary with the Augmented Dickey-Fuller (ADF) test [42]. The ADF tests the null hypothesis that the series is non-stationary. When the ADF is applied to demanded power data by the hotel recorded during a year, the test gives a *p*-value < 0.01, meaning that the demanded power data along a year is stationary. Therefore, the difference in the ARIMAX will be d=0, *ARIMAX(p,0,q)*. However, the time series has a seasonal trend with a period of 24 h (to see Figure 2A,B) and the Auto-Correlation Function (ACF) and the Partial Auto-Correlation Function (PACF) keep oscillating outside the significance limits, indicating that the correlations are significantly different from zero. If we eliminate this component of seasonality to 24 h, we would obtain the values that are not predictable and the ACF and the PACF would stay within the significance bounds. However, in the present work, we want to reproduce all the behavior of the total power demand in each hour using an ARIMAX model as reference model to compare the hybrid AI model developed in this research.

The *auto.arima()* function in R software is used to find the parameters *p* and *q* for the ARIMAX model [43]. The *auto.arima()* function uses a variation of the Hyndman–Khandakar algorithm [44,45], which combines unit root tests, minimization of the Akaike Information Criteria (AIC) to obtain an ARIMAX model (*ARIMAX(p,d,q)*).

To forecast the demand power of our hotel for the next 24 h, the ARIMAX model is trained with the time series of the previous 31 days. To validate the models, we select five validation days taking into account two different reasons: random dates (3 January and 29 September), and special dates for tourists (11 February, 1 August, and 31 December). These special dates are New Year Eve, Carnival week, and holidays; the occupancy should be affected by these dates but we do not select the days based on occupancy change.

Finally, the Ljung-Box test statistic is computed over the training data to examine the null hypothesis of independence of the residuals (the null hypothesis of the Ljung–Box test is that the model does not show lack of fit). The statistic is based on $n_{lag}$ auto-correlation coefficients and the number of degrees of freedom is fixed to h=1, as $h=n_{lag}-\left(p+q\right)$, then $n_{lag}=p+q+1$. The *p*-values obtained are less than 0.05, therefore we reject the null hypothesis, which means that the models show lack of fit (see Table 1).

### 3.3. Bagged Decision Trees Modeling

Regression and classification trees are a class of powerful algorithms for machine learning that allows for a high accuracy and are characterized by their clarity of information representation [46]. Basically, the basic mechanism of a decision tree consists of splitting a data set into smaller subsets according to specific features of the data. As a result, a set of decision-making rules are obtained that model the input data. While classification trees focus on classification of data into categories, regression trees focus on predicting numeric values.

Decision trees use a tree-like structure based on nodes and leaves to make predictions. The growth of this structure is done by evaluating features on the input data. Thus, three different types of nodes are distinguished:

- Root node: this node has no incoming edges and several (or zero) outgoing edges.
- Internal node: characterized by one incoming edge and two or more outgoing edges.
- Leaf: it has one incoming edge and no outgoing edges.

In general, the performance of a single decision tree is limited. That is why their structure is often improved by creating an ensemble of them and collecting their predictions. The main idea behind the ensemble model is that a group of individual learners join to form a more accurate learner.

One of the most efficient ensembles proposed for decision trees is based on Bootstrap Aggregation [47]. They are called bagged trees and are used with the aim of reducing the variance of a decision tree. The main idea behind bagged trees is to create several subsets of data of the same size extracted from the training sample. Common methods for this are disjoint partitions, small bags, no replication small bags, and disjoint bags. Each subset is used to train a decision tree. This process will generate an ensemble of different models. Finally, a global predictor with better performance is defined as the aggregation of the local predictors.

### 3.4. Hybrid Intelligent Modeling

**Figure 3:**
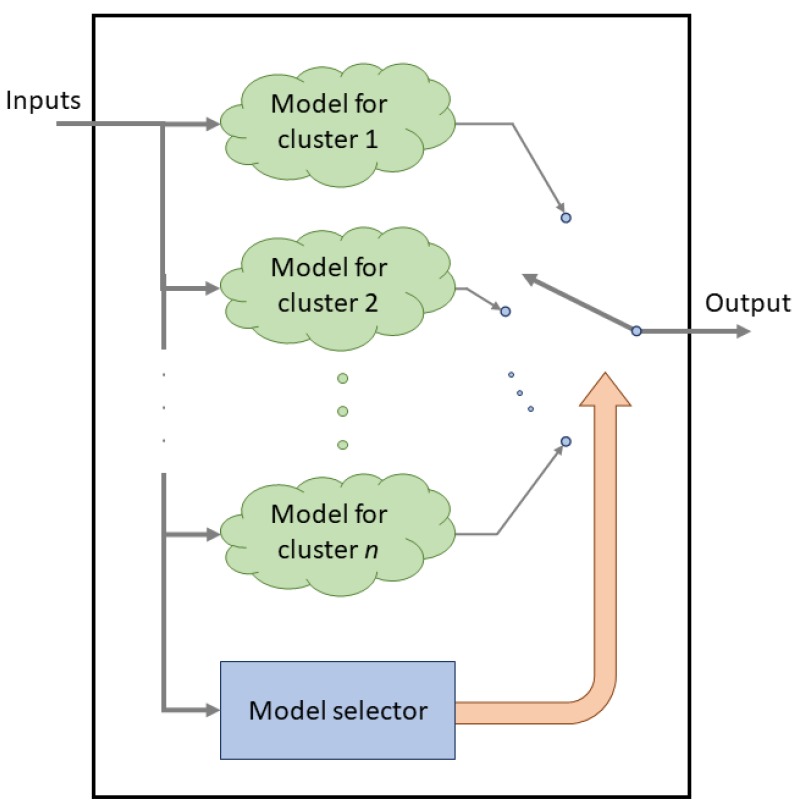
Internal layout for a hybrid model.

A Hybrid Intelligent model allows improvement of the global performance of a model. These types of models include a clustering and a regression phase; when the model was trained, the inputs are used to select a specific regression model to calculate the output. The internal structure of a hybrid model is shown in Figure 3. The internal regression models for each cluster could be any type of regression algorithm. In this work ANN and Support Vector Machine for Regression were used.

#### 3.4.1. K-Means Clustering Algorithm

The K-Means algorithm is a well-known unsupervised technique used to create groups of data [48,49]. The clusters contain data with similar characteristics among them. In our case, the algorithm is trained to minimize an error function based on the centroid Euclidean distance.

The K-Means algorithm starts with the selection of the number of clusters and then a centroid for each cluster is randomly assigned. The training iterates the next steps until the data do not change its cluster, compared with the previous iteration. The method can be summarized in these two steps (after the initialization):

- Determine the cluster for each sample data based on the distance to the centroids. The cluster assigned should be the closest one to the sample.
- Calculate new centroids as the center of the clusters, taking into account all the samples per cluster.

When the cluster assigned to each sample and the centroids are the same, it means that the centroid calculation is finished. Then, the K-Means algorithm is ready to be used with new data. Once the final centroids are obtained, they can be used to assign new data to its cluster. This algorithm obtains the best results when the data are well distributed in hyperspace, and the cluster data has a hypersphere shape [49].

#### 3.4.2. Multi-Layer Perceptron

The Multi-Layer Perceptron (MLP) is one of the most typical configurations of an ANN. It is a feedforward ANN with one input layer, one or more hidden layers, and one output layer [50,51,52]. Each layer has several neurons that depends on the number of inputs (in the case of input layer), the configuration of the MLP (for the hidden layers), and the number of outputs (for the output layer).

All the neurons in a layer have the same inputs that would be the input data in the case of the first layer, and the output of the previous layer in other case. Each neuron input has a specific weight, and all of them are added before calculating the output of the activation function that would be the output of the neuron. There are some typical activation functions such as Tan-Sigmoid, Log-Sigmoid, Linear, Step, etc., each for a specific application. For regression, the usual ones are the Tan-Sigmoid for the neurons in all layers, except for the output layer that used to have a linear function. The Tan-Sigmoid activation function is shown in Equation (1).

(1) $$F\left(t\right)=\frac{e^{t}-e^{-t}}{e^{t}+e^{-t}}$$

In the training phase, the weight for each internal signal is adjusted based on the desired output [53]. The output of the whole ANN is calculated based on Equation (2), where the adjusted parameters are in the θ variable.

(2) $$f_{\theta }\left(x\right)=\beta +\sum_{i=1}^{k}a_{i}\phi \left(w_{i}^{T}x+b_{i}\right)$$

where:

$x={\left(x\left(1\right),\ldots ,x\left(d\right)\right)}^{T}\in {ℜ}^{d}$ is the inputs vector
*k* is the hidden layers number
ϕ is a bounded transfer function
$\theta =(\beta ,a_{1},\ldots ,a_{k},b_{1},\ldots ,b_{k},w_{11},\ldots ,w_{kd})$ is the model parameter vector
$w_{i}={\left(w_{i1},\ldots ,w_{id}\right)}^{T}\in {ℜ}^{d}$ is the parameter vector for the hidden unit *i*

#### 3.4.3. Support Vector Machines for Regression

Support Vector Machines (SVM) is a supervised machine-learning technique that is commonly used for classification problems. SVM can also be used for regression (SVR) by performing minor changes in the original algorithm used for classification problems. The Support Vector Regression is based on a nonlinear mapping to transform the data in a new high-dimensional space and, then, a linear regression is applied to calculate the desired output. In this work, a modification of SVR, called Least Squares SVR (LS-SVR), will be applied. LS-SVR uses the Least Squares to minimize the objective function [54,55,56,57,58].

In this case, a system of linear equations is solved with the aim of obtaining an approximation of the solution, which is not achieving the optimal solution that we would get with the original SVR algorithm. Nevertheless, this modification provides a comparable generalization performance as the SVR [59,60]. LS-SVR is the given name of the application of LS-SVM to regression [57,58].

A classical squared loss function is used instead of the insensitive loss function when the LS-SVR is applied. With this change, a linear KarushKuhn–Tucker is solved to build the Lagrangian (Equation (3)).

(3) $$\left[\begin{array}{cc} 0 & I_{n}^{T}\\ I_{n} & K+\gamma_{-1}I \end{array}\right]\left[\begin{array}{c} b_{0}\\ b \end{array}\right]=\left[\begin{array}{c} 0\\ y \end{array}\right]$$

where:

$I_{n}$ is a vector of *n* ones
*T* means transpose of a matrix or vector
γ a weight vector
*b* regression vector
$b_{0}$ is the model offset

The weight vector (γ) as well as the width of the kernel (σ) are the only variables needed in LS-SVR [58].

## 4. An Intelligent Model for Power Demand

**Figure 4:**
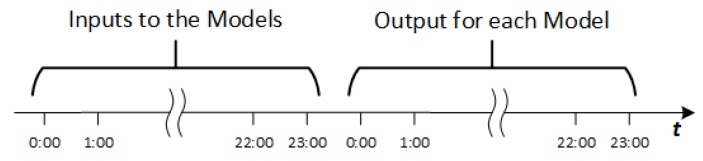
Input and output variables in time distribution.

The aim of this work is to synthesize a model to predict the hotel power consumption in a 24 h horizon. Instead of using only one model, the proposal is based on 24 hybrid models that calculate the consumption at each hour for the next day. Figure 4 represents the inputs and outputs in a time scale. As can be observed, the output of the model is calculated with the previous 24 h values.

**Figure 5:**
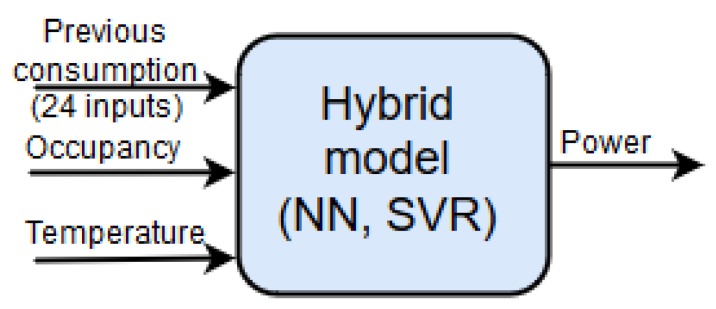
Hybrid model to predict the next day power.

Moreover, as shown in Figure 5, the daily mean temperature and the occupation are used as inputs to improve the accuracy of the prediction. The prediction along the 24 h horizon is done by using 24 hybrid models like this. Each model will provide a prediction of one hour in the next day.

As mentioned before, the model was trained with data of one year (electricity demand, occupancy, and mean daily temperature). The number of days used for training was 360. To validate the model, 5 days distributed along the whole year were used. The training procedure is divided in three phases:

1. Clustering training. This phase is the same for all the hybrid models, as they share the same inputs.
2. Regression training. For each cluster, two different regression algorithms (MLP and SVR) were evaluated. In the case of ANN, different internal configurations were considered.
3. Performance calculation. As the number of clusters for each model is not a predefined, it is necessary to calculate the best cluster assignment based on the achieved error.

As shown in Figure 3, each of the 24 submodels has a model selector to activate the local model according to the inputs. The model selector is based on the minimization of the Euclidean distance to each of the centroids of the clusters. In this way, the cluster with a lower distance to the input data is activated.

### 4.1. Clustering Training

As mentioned earlier, K-means was used for clustering. The maximum cluster number was set to 10. The method was programmed to stop when the resulting cluster had fewer than 15 samples. The algorithm resulted in 7 different clusters. Two configurations were considered: a global model (with all the data), and 6 local models (dividing the data from 2 to 7 groups). Each training was repeated 20 times with random initial centroids, to ensure that the algorithm obtain the best results. In Table 2 an example of the cluster number samples is shown.

### 4.2. Regression Training

**Figure 6:**
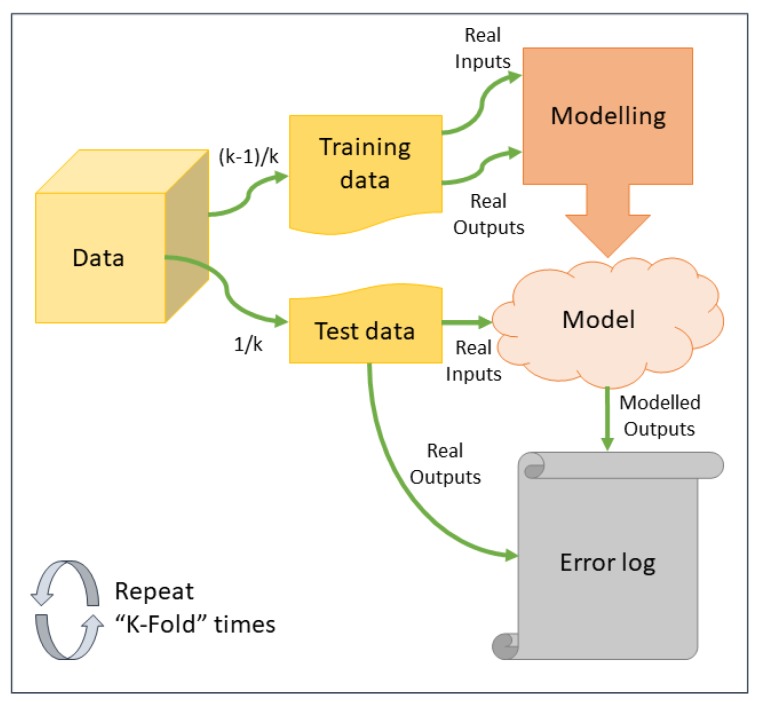
Cross validation using K-Fold.

The training of the regression algorithm was made using K-Fold cross validation. Figure 6 shows the training phase generally. This step is repeated 16 times for each cluster; 15 different configurations of the MLP (with different neurons number in the hidden layer) and LS-SVR. The errors obtained after training each regression technique are stored and compared when all the algorithms are trained for a specific cluster. In this the way, the best algorithm per cluster is selected.

The several MLP configurations are tested, all of them with Tan-Sigmoid activation function for the internal neurons (in the hidden layer); and, in the output layer, a Linear activation function is set. The difference in the configurations were the quantity of hidden layer neurons. This layer size varied from 1 to 15 neurons. To train each MLP configuration, the Levenberg–Marquardt algorithm was used. Moreover, to finish the training phase, gradient descent was used based on the error.

The auto-tuning functions implemented in the MATLAB toolbox by KULeuven-ESAT-SCD are used to train the LS-SVR algorithm. As this toolbox has the classification and the regression type for Support Vector Machines, it is necessary to set the type to @Function Estimation@. The model kernel was configured as Radial Basis Function (RBF) and the cost criteria is @leaveoneoutlssvm@.

To illustrate the procedure, Table 3 shows the best regression algorithm for each cluster in one of the hybrid models. All of them are different as they have different setups (in the case of MLP) and different adjusted internal parameters (in both techniques).

### 4.3. Hybrid Model Performance Calculation

To calculate the best hybrid configuration in the models, the Mean Squared Error (MSE) was used as a measurement of the error in each cluster. This performance measure is used to select the best algorithm. To represent the error of a hybrid configuration, the MSE and the number of samples in each cluster were taken into account to calculate the pondered error. Table 4 shows an example of the resulting MSE for a specific model (for prediction of power at 18:00). In this table, the MSE for each cluster in the different hybrid configuration is shown. In this specific case, the best configuration is achieved with 2 clusters in the hybrid model.

## 5. Results and Discussion

A hybrid intelligent model was obtained using the data of a luxury hotel. The model has three inputs: the energy demand in the previous 24 h, the mean temperature of the previous day and the occupancy rate of the hotel. The model will provide hourly energy demand for the next 24 h. The training of the model was done using data from 360 days and the remaining days (5) were for validation purposes. These days were chosen to be distributed along the year: 31 December, 3 January, 11 August, and 29 September.

The model is divided in 24 submodels, each one predicting the load in a specific hour of the next day. The setup of the 24 internal models used regression models based on LS-SVR or ANN. The ANN are MLP with one hidden layer of neurons and Tan-Sigmoid as the activation function. In the output layer, as the MLP is used for regression, the linear activation function is selected. The MLP training algorithm used was Levenberg–Marquardt. Up to 15 different configurations were considered for the ANN and LS-SVR. As commented, K-Fold cross validation was used to train the model. This procedure was repeated 16 times for each cluster.

**Figure 7:**
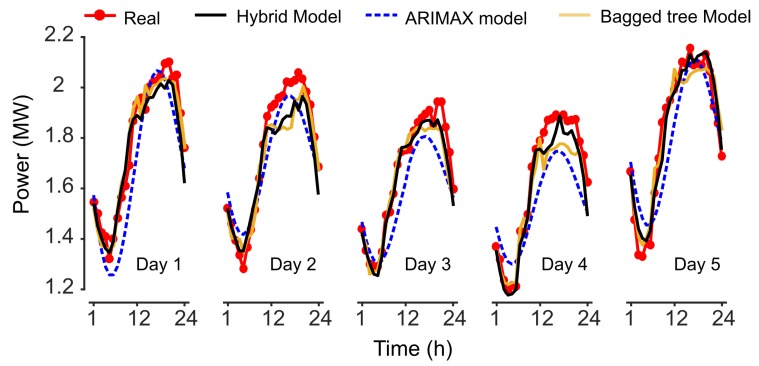
Predicted power demand by the model. Validation of the hybrid intelligent model, the ARIMAX model and the bagged decision tree model.

The 24 h forecast of the five validation days using the hybrid model is shown in Figure 7 (solid line). It is remarkable how the forecast follows satisfactorily the real load curve. The study includes a comparison with an ARIMAX prediction method that is a technique for short-term load prediction. Also, a comparison with an advanced prediction method based on bagged decision trees is included. Both methods present an acceptable behavior with low error indexes. ARIMAX offers a higher error index while bagged decision tree, as expected, presents a much better mean square error. However, when compared to the hybrid proposal of this paper, as can be observed, the hybrid model overperforms clearly the ARIMAX proposal and performs better than the bagged decision tree method.

In Table 5 a detailed performance evaluation is done. The accuracy of the model was assessed by evaluating the Mean Absolute Percentage Error (MAPE), the Mean Absolute Error (MAE), the MSE, and the Maximum Error (Max.). As can be seen, all the error indexes are better for the hybrid model. Thus, the average of MAE of the five-day data set is around $49.51\times 10^{-3}$ MW for hybrid model compared to $106.36\times 10^{-3}$ for the ARIMAX model and $55.18\times 10^{-3}$ for the bagged tree method.

Considering that the power base is the contracted power of the hotel (1.84 MW), the normalized averaged MAE is 0.0141 (2.69%). This means the intelligent model proposed can predict the power load for the next 24 hours with an accuracy close to 97.31%. This value is higher than the accuracy obtained for the ARIMAX and for the bagged decision tree method. With the ARIMAX model the normalized average MAE is 5.78% with an accuracy of around 94.22% which is clearly lower than the results of the hybrid model. For the bagged tree algorithm, the resulting normalized average MAE is 3.00% with an accuracy of 97.00%. As observed, the proposed method involves an improvement compared to the other methods in the overall performance of the predictor.

It is important to observe that the ARIMAX model is obtained for each prediction day using information from the previous 31 days. In this sense, the ARIMAX can have some adaptation according to the previous variations of the data. This implies that the real implementation of this method involves some online computational effort to obtain the model. In most cases this fact will not be relevant, but for some embedded applications with low cost equipment, it is necessary to take it into consideration. On the other hand, the hybrid intelligent model proposed is an explicit model that is completely obtained offline using data from the whole year. In this sense, the online computational load and storage is reduced.

## 6. Conclusions

This work proposes an intelligent hybrid model to predict the short-term energy demand in a hotel. The model predicts the power load of the hotel for each hour in a 24-h horizon. The model uses information from the previous energy demand of the hotel, the mean temperature of the previous day, and the occupancy rate of the resort.

The solution proposes an overall model decomposed in 24 submodels, each one predicting the energy demand for each hour of the next day. Its structure is based on AI techniques. Three main techniques were used: clustering, to define the local models, and MLP and LS-SVR, to implement the model corresponding to the clusters.

The results obtained show that the proposed model was able to satisfactorily predict the energy load for the next 24 h. A comparison with the real curve results in a mean absolute error of 0.04951 MW. This error must be evaluated, taking into account the high overall power demand. The value of the mean absolute error percentage normalized by 1.84 MW (contracted power of the hotel) is 2.69% which attests to the accuracy of the proposed model. The results were compared with a conventional forecast technique based on ARIMAX modeling and an advanced method based on bagged decision trees. The hybrid model obtained better results than those obtained by the ARIMAX model and bagged tree models (5.78% and 3.00% mean absolute error, respectively) and averaged maximum error normalized, respectively)).

The proposed model appears to be a potential tool to improve the prediction of power demand in hotels. Energy management in the hotel where the study took place is based on a prediction model that uses linear correlation by least squares. In this model, the consumption (energy daily prediction, EDP) is directly proportional to the Cooling Degree Days (CDD), calculated by BizEE Software Limited, and occupancy. Thus, the new proposal presented offers a great potential to improve the accuracy of the existing prediction method in the hotel. The availability of these predictions plays an important role in increasing the efficiency of energy management systems with the aim of reducing the energy costs and guaranteeing environmental sustainability. 

## Figures and Tables

**Table 1:** Errors achieve by the ARIMAX models for training set, 31 days before the selected day.

	ARIMAX	MAPE (%)	Box-Ljung Test, *p*-Value
Day 1 (31 December)	ARIMAX(4,0,1)	2.55	2.7 $\times \;10^{-5}$
Day 2 (3 January)	ARIMAX(4,0,1)	2.51	1.2 $\times \;10^{-7}$
Day 3 (11 February)	ARIMAX(4,0,1)	2.43	1.1 $\times \;10^{-5}$
Day 4 (1 August)	ARIMAX(4,0,1)	3.22	9.5 $\times \;10^{-7}$
Day 5 (29 September)	ARIMAX(3,0,1)	2.88	5.4 $\times \;10^{-2}$

**Table 2:** Number of samples per cluster in the model to predict the power at 15:00.

	Cl-1	Cl-2	Cl-3	Cl-4	Cl-5	Cl-6	Cl-7
Global	354						
Hybrid 2	147	207					
Hybrid 3	91	91	172				
Hybrid 4	52	65	97	140			
Hybrid 5	40	43	65	87	119		
Hybrid 6	36	43	59	60	76	80	
Hybrid 7	21	39	47	57	60	61	69

**Table 3:** Best algorithm per cluster in the model to predict the power at 9:00.

	Cl-1	Cl-2	Cl-3	Cl-4	Cl-5	Cl-6	Cl-7
Global	LS-SVR						
Hybrid 2	LS-SVR	LS-SVR					
Hybrid 3	MLP-11	LS-SVR	LS-SVR				
Hybrid 4	MLP-15	LS-SVR	LS-SVR	LS-SVR			
Hybrid 5	LS-SVR	LS-SVR	LS-SVR	LS-SVR	LS-SVR		
Hybrid 6	MLP-15	MLP-14	MLP-12	MLP-11	MLP-12	LS-SVR	
Hybrid 7	LS-SVR	MLP-14	LS-SVR	MLP-13	MLP-13	MLP-11	LS-SVR

**Table 4:** MSE per cluster in the model to predict the power at 18:00.

	Cl-1	Cl-2	Cl-3	Cl-4	Cl-5	Cl-6	Cl-7	Hybrid MSE
Global	0.9454							0.9454
Hybrid 2	0.8620	0.7596						0.8021
Hybrid 3	1.0792	1.3943	0.8693					1.0582
Hybrid 4	2.5665	0.8984	0.9411	0.7035				1.0780
Hybrid 5	1.2082	2.1560	1.0623	0.9041	0.8080			1.0873
Hybrid 6	1.0036	2.3759	1.1940	0.5146	1.3799	0.9011		1.1768
Hybrid 7	0.8151	2.2792	1.4413	0.5952	1.6359	1.6617	1.6345	1.4689

**Table 5:** Errors achieve by the models. (Mean Absolute Percentage Error (MAPE), the Mean Absolute Error (MAE), the Mean Square Error (MSE), and the Maximum Error (Max.)).

	ARIMAX Model			Bagged Tree Model			Hybrid Model		
	MAPE (%)	MAE ($10^{-3}$ MW)	MSE ($10^{-3}$ MW${}^{2}$)	MAPE (%)	MAE ($10^{-3}$ MW)	MSE ($10^{-3}$ MW${}^{2}$)	MAPE (%)	MAE ($10^{-3}$ MW)	MSE ($10^{-3}$ MW${}^{2}$)
Day 1	6.27	107.73	16,402	2.70	46.73	3167	2.98	53.94	4515
Day 2	4.73	82.80	9027	3.53	64.05	6473	3.74	68.36	6661
Day 3	6.01	103.46	14,832	2.43	41.88	2944	1.97	33.75	2052
Day 4	8.36	138.87	21,808	4.02	70.24	7388	2.96	49.76	3773
Day 5	5.73	98.93	15,173	2.98	52.99	4015	2.43	41.76	3035
Mean	6.22	106.36	15,449	3.13	55.18	4797	2.81	49.51	4007

## References

[1] Pieri S.P., Tzouvadakis I., Santamouris M. (2015). Identifying energy consumption patterns in the Attica hotel sector using cluster analysis techniques with the aim of reducing hotels’ CO_2_ footprint. Energy Build..

[2] Dalton G.J., Lockington D.A., Baldock T.E. (2009). Feasibility analysis of renewable energy supply options for a grid-connected large hotel. Renew. Energy.

[3] Deng S.M., Burnett J. (2000). Study of energy performance of hotel buildings in Hong Kong. Energy Build..

[4] Papamarcou M., Kalogirou S. (2001). Financial appraisal of a combined heat and power system for a hotel in Cyprus. Energy Convers. Manag..

[5] Priyadarsini R., Xuchao W., Eang L.S. (2009). A study on energy performance of hotel buildings in Singapore. Energy Build..

[6] Cabello Eras J., Sousa Santos V., Sagastume Gutiérrez A., Guerra Plasencia M., Haeseldonckx D., Vandecasteele C. (2016). Tools to improve forecasting and control of the electricity consumption in hotels. J. Clean. Prod..

[7] Hilton Worldwide (2018). Energy. http://cr.hiltonworldwide.com/download/Hilton{_}CRReport{_}Energy.pdf.

[8] Atique S., Noureen S., Roy V., Subburaj V., Bayne S., Macfie J. Forecasting of total daily solar energy generation using ARIMA: A case study. Proceedings of the 2019 IEEE 9th Annual Computing and Communication Workshop and Conference (CCWC).

[9] Mat Daut M.A., Hassan M.Y., Abdullah H., Rahman H.A., Abdullah M.P., Hussin F. (2017). Building electrical energy consumption forecasting analysis using conventional and artificial intelligence methods: A review. Renew. Sustain. Energy Rev..

[10] Nguyen H., Hansen C.K. Short-term electricity load forecasting with Time Series Analysis. Proceedings of the 2017 IEEE International Conference on Prognostics and Health Management (ICPHM).

[11] Suganthi L., Samuel A.A. (2012). Energy models for demand forecasting: A review. Renew. Sustain. Energy Rev..

[12] Singh A.K., Khatoon S. (2013). An Overview of Electricity Demand Forecasting Techniques. Netw. Complex Syst..

[13] Shao Z., Chao F., Yang S.L., Zhou K.L. (2017). A review of the decomposition methodology for extracting and identifying the fluctuation characteristics in electricity demand forecasting. Renew. Sustain. Energy Rev..

[14] Khosravani H., Castilla M., Berenguel M., Ruano A., Ferreira P. (2016). A Comparison of Energy Consumption Prediction Models Based on Neural Networks of a Bioclimatic Building. Energies.

[15] Torres J.M., Aguilar R., Aguilar R.M., Zúñiga K.V. (2018). Deep Learning to Predict the Generation of a Wind Farm. J. Renew. Sustain. Energy.

[16] Jove E., Gonzalez-Cava J.M., Casteleiro-Roca J.L., Pérez J.A.M., Calvo-Rolle J.L., de Cos Juez F.J. An intelligent model to predict ANI in patients undergoing general anesthesia. Proceedings of the International Joint Conference SOCO’17-CISIS’17-ICEUTE’17.

[17] Jove E., Gonzalez-Cava J.M., Casteleiro-Roca J.L., Méndez-Pérez J.A., Antonio Reboso-Morales J., Javier Pérez-Castelo F., Javier de Cos Juez F., Luis Calvo-Rolle J. (2018). Modelling the hypnotic patient response in general anaesthesia using intelligent models. Log. J. IGPL.

[18] Casteleiro-Roca J.L., Jove E., Gonzalez-Cava J.M., Pérez J.A.M., Calvo-Rolle J.L., Alvarez F.B. (2018). Hybrid model for the ANI index prediction using Remifentanil drug and EMG signal. Neural Computing and Applications.

[19] Jove E., Casteleiro-Roca J.L., Quintián H., Méndez-Pérez J.A., Calvo-Rolle J.L. (2019). A fault detection system based on unsupervised techniques for industrial control loops. Expert Systems.

[20] Neto A.H., Fiorelli F.A.S. (2008). Comparison between detailed model simulation and artificial neural network for forecasting building energy consumption. Energy Build..

[21] Muralitharan K., Sakthivel R., Vishnuvarthan R. (2018). Neural network based optimization approach for energy demand prediction in smart grid. Neurocomputing.

[22] Zúñiga K.V., Castilla I., Aguilar R.M. (2014). Using fuzzy logic to model the behavior of residential electrical utility customers. Appl. Energy.

[23] Abreu T., Alves U.N., Minussi C.R., Lotufo A.D.P., Lopes M.L.M. Residential electric load curve profile based on fuzzy systems. Proceedings of the 2015 IEEE PES Innovative Smart Grid Technologies Latin America (ISGT LATAM).

[24] Bedi J., Toshniwal D. (2019). Deep learning framework to forecast electricity demand. Appl. Energy.

[25] Wasseem Ahmad M., Mourad A., Rezgui Y., Mourshed M. (2019). Deep Highway Networks and Tree-Based Building Energy Consumption. Energies.

[26] Chen Y., Tan H. (2017). Short-term prediction of electric demand in building sector via hybrid support vector regression. Appl. Energy.

[27] Seyedzadeh S., Rahimian F.P., Glesk I., Roper M. (2018). Machine learning for estimation of building energy consumption and performance: A review. Vis. Eng..

[28] Jove E., Blanco-Rodríguez P., Casteleiro-Roca J.L., Moreno-Arboleda J., López-Vázquez J.A., de Cos Juez F.J., Calvo-Rolle J.L. (2017). Attempts prediction by missing data imputation in engineering degree. Proceedings of the International Joint Conference SOCO’17-CISIS’17-ICEUTE’17.

[29] Gonzalez-Cava J.M., Reboso J.A., Casteleiro-Roca J.L., Calvo-Rolle J.L., Méndez Pérez J.A. (2018). A novel fuzzy algorithm to introduce new variables in the drug supply decision-making process in medicine. Complexity.

[30] Casteleiro-Roca J.L., Barragán A.J., Segura F., Calvo-Rolle J.L., Andújar J.M. (2019). Fuel Cell Output Current Prediction with a Hybrid Intelligent System. Complexity.

[31] Casteleiro-Roca J.L., Perez J.A.M., Piñón-Pazos A.J., Calvo-Rolle J.L., Corchado E. (2019). Intelligent Model for Electromyogram (EMG) Signal Prediction During Anesthesia. J. Mult. Valued Log. Soft Comput..

[32] Olatomiwa L., Mekhilef S., Ismail M., Moghavvemi M. (2016). Energy management strategies in hybrid renewable energy systems: A review. Renew. Sustain. Energy Rev..

[33] Comodi G., Renzi M., Cioccolanti L., Caresana F., Pelagalli L. (2015). Hybrid system with micro gas turbine and PV (photovoltaic) plant: Guidelines for sizing and management strategies. Energy.

[34] Serale G., Fiorentini M., Capozzoli A., Bernardini D., Bemporad A. (2018). Model Predictive Control (MPC) for Enhancing Building and HVAC System Energy Efficiency: Problem Formulation, Applications and Opportunities. Energies.

[35] Jo H., Yoon Y. (2018). Intelligent smart home energy efficiency model using artificial TensorFlow engine. Hum.-Centric Comput. Inf. Sci..

[36] Ruban A.A.M., Rajasekaran G.M., Pasupathi T., Rajeswari N. A fuzzy-logic based management system in smart-microgrid for residential applications. Proceedings of the 2016 International Conference on Emerging Trends in Engineering, Technology and Science (ICETETS).

[37] Mardani A., Zavadskas E., Streimikiene D., Jusoh A., Nor K., Khoshnoudi M. (2016). Using fuzzy multiple criteria decision making approaches for evaluating energy saving technologies and solutions in five star hotels: A new hierarchical framework. Energy.

[38] Acosta A., González A., Zamarreño J., Álvarez V. (2016). Energy savings and guaranteed thermal comfort in hotel rooms through nonlinear model predictive controllers. Energy Build..

[39] Vega Lara B., Castellanos Molina L., Monteagudo Yanes J., Rodríguez Borroto M. (2016). Offset-free model predictive control for an energy efficient tropical island hotel. Energy Build..

[40] Riverón I., Gómez J.F., González B., Méndez J.A. (2019). An intelligent strategy for hybrid energy system management. Renew. Energy Power Qual. J..

[41] EXCELTUR (2017). EXCELTUR, Alliance for Excellency in Tourism. http://www.exceltur.org/exceltur-in-english/.

[42] Fuller W.A. (1996). Introduction to Statistical Time Series.

[43] Hyndman R. Auto.Arima Function from Forescast v8.6 | R Documentation. https://otexts.com/fpp2/arima-r.html.

[44] Hyndman R.J., Khandakar Y. (2008). Automatic Time Series Forecasting: The forecast Package for R. J. Stat. Softw..

[45] Wang X., Smith K., Hyndman R. (2006). Characteristic-Based Clustering for Time Series Data. Data Min. Knowl. Discov..

[46] Wei-Yin L. (2011). Classification and regression trees. Wiley Interdiscip. Rev. Data Min. Knowl. Discov..

[47] Breiman L. (1994). Bagging Predictors: Technical Report No. 421.

[48] Qin A., Suganthan P. (2005). Enhanced neural gas network for prototype-based clustering. Pattern Recogn..

[49] Kaski S., Sinkkonen J., Klami A. (2005). Discriminative clustering. Neurocomputing.

[50] Wasserman P. (1993). Advanced Methods in Neural Computing.

[51] Zeng Z., Wang J. (2010). Advances in Neural Network Research and Applications.

[52] Osborn J., Guzman D., de Cos Juez F.J., Basden A.G., Morris T.J., Gendron E., Butterley T., Myers R.M., Guesalaga A., Sanchez Lasheras F. (2014). Open-loop tomography with artificial neural networks on CANARY: on-sky results. Mon. Not. R. Astron. Soc..

[53] Rynkiewicz J. (2012). General bound of overfitting for MLP regression models. Neurocomputing.

[54] Cristianini N., Shawe-Taylor J. (2000). An Introduction to Support Vector Machines and Other kernel-Based Learning Methods.

[55] Vilán Vilán J.A., Alonso Fernández J.R., García Nieto P.J., Sánchez Lasheras F., de Cos Juez F.J., Díaz Muñiz C. (2013). Support Vector Machines and Multilayer Perceptron Networks Used to Evaluate the Cyanotoxins Presence from Experimental Cyanobacteria Concentrations in the Trasona Reservoir (Northern Spain). Water Resour. Manag..

[56] Wang R., Wang A., Song Q. (2012). Research on the alkalinity of sintering process based on LS-SVM Algorithms. Advances in Computer Science and Information Engineering.

[57] Guo Y., Li X., Bai G., Ma J. Time Series Prediction Method Based on LS-SVR with Modified Gaussian RBF. Proceedings of the International Conference on Neural Information Processing.

[58] Wang L., Wu J. (2012). Neural network ensemble model using PPR and LS-SVR for stock et eorecasting. International Conference on Intelligent Computing.

[59] Steinwart I., Christmann A. (2008). Support Vector Machines.

[60] Vapnik V. (1995). The Nature of Statistical Learning Theory.

